# A Case-Control Study of the Factors Associated with Anemia in Chinese Children Aged 3–7 years Old

**DOI:** 10.1155/2023/8316658

**Published:** 2023-03-20

**Authors:** Jinsong Mou, Haishan Zhou, Zhangui Feng, Shiya Huang, Zhaohui Wang, Chaoyu Zhang, Yudong Wang

**Affiliations:** Pingshan District Maternal & Child Healthcare Hospital of Shenzhen, Pingshan General Hospital of Southern Medical University, Shenzhen, China

## Abstract

**Background:**

Anemia in children is still an important public problem in China and can have a profound impact on the physical and mental health of children. The purpose of this study was to explore the risk factors for anemia among Chinese children aged 3–7 years old and to provide some basis for the prevention and control of anemia.

**Methods:**

A matched case-control study was conducted and 1104 children (552 cases and 552 controls) were recruited in this study. Cases were children who were diagnosed with anemia by the doctor of physical examination and checked by one deputy chief physician of pediatrics, and controls were healthy children without anemia. Data were collected using a self-designed structured questionnaire. Univariable and multivariable analyses were used to identify independent determinants of anemia. *P* values less than 0.05 were used to declare statistical significance.

**Results:**

In the multivariable analyses, maternal anemia before or during pregnancy and lactation (OR = 2.14, 95% CI: 1.10∼4.15; OR = 2.86, 95% CI: 1.66∼4.94; OR = 2.51, 95% CI: 1.13∼5.60), gestational weeks (OR = 0.72, 95% CI: 0.53∼0.96), having G6PD deficiency or thalassemia (OR = 8.12, 95% CI: 2.00∼33.04; OR = 36.25, 95% CI: 10.40∼126.43), having cold and cough in previous two weeks (OR = 1.56, 95% CI: 1.04∼2.34), family income (OR = 0.80, 95% CI: 0.65∼0.97), and being a picky eater (OR = 1.80, 95% CI: 1.20∼2.71) were determinants of anemia in children aged 3–7 years old.

**Conclusions:**

Some of the identified factors are modifiable and could be targeted to reduce childhood anemia. More emphasis should be given by the concerned bodies to intervene in the anemia problem by improving the maternal health education, screening for disease-related anemia, requesting medical services in a timely manner, improving the economic status of households, promoting dietary habits, and improving sanitation and hygiene practices.

## 1. Introduction

Anemia is regarded as a decrease in the number of red blood cells or their oxygen-carrying capacity. It is also defined as a hemoglobin level below 11 mg/dl for children 6 to 59 months of age [1]. Childhood anemia is a major public health problem worldwide affecting both developing and developed countries [2]. It is associated with adverse effects including impaired immunity and, cognitive development and reduce capacity for work [3–5].

Anemia is a global health problem. In 2008, the World Health Organization (WHO) reported the global anemia prevalence was 47.4% (95% confidence interval [CI] 45.7–49.1) in preschool-age children and 25.4% (95% CI 19.9–30.9) in school-age children. In 2011, one paper reported that 293 million (47%) children younger than 5 years were affected by anemia [6]. In China, the researchers have reported the prevalence of anemia among children between 6% and 27% from 2009 to 2019 [7–10].

The causes of anemia are multifactorial, including shortage of hematopoietic materials [11]. Some reviews have reported the factors related to anemia, such as poor dietary diversity, food insecurity, deworming, maternal anemia, gastrointestinal disease, and educational status [12, 13].

Children are deemed to be at greater risk than other populations [14]. Over the recent years, more attention has been on children. However, many studies related to anemia in children have been cross-sectional, and few analytical studies have been reported. Pingshan is one district of Shenzhen municipality located in the northeast. The economic level is relatively lower, and the migrant population is relatively large. Implementing concrete strategies to reduce is a major task. Therefore, it is important to identify the associated factors to address the problem. To explore factors related to anemia in children, a case-control study was conducted, which could provide support for future anemia prevention and control measures.

## 2. Methods

### 2.1. Study Design and Participants

A case-control study was conducted from 2018 to 2020 among children aged 3–7 years old in all kindergartens of Pingshan District, Shenzhen. Every child received physical examination and blood tests at least once a year. All children identified as having anemia by physical examination and HemoCue were included in the case-control study as cases. Then healthy children without anemia were selected as controls from the same class. A case/control rate of 1 : 1 was applied. The matching conditions were the same sex and an age difference of less than 3 months. The sample size was determined by the software of PASS. Assuming a proportion of exposure in cases was 12.0%, a power of 80% and 5% of significance, and odds ratio being assumed to be 1.7, we required 528 cases and 528 controls (1 control per 1 case). In our study, we finally included 552 cases and 552 controls.

### 2.2. Measurements

Hemoglobin concentration of children was measured using the HemoCue. The operational instructions were strictly obeyed and the outcomes were identified by multiple researchers. Anemia was defined as hemoglobin <110 g/L for children under 59 months or <115 g/L for children over 5 years. Weight and height were measured for all children. The height and weight of children were measured by intelligent physical examination instrument for children (Kangwa Intelligent physical examination instrument). The scales were calibrated each morning and checked at regular intervals throughout the day. All measurements were made by a highly trained research anthropometrist. In addition, a repeated measurement for 10% of the children randomly selected each day was conducted. If the two measurements differed by more than 0.5 cm, a third measurement was taken. When the two measurements were similar, their mean was calculated. Weight-for-age (WAZ), height-for-age (HAZ), and BMI-for-age (BMIZ) z-scores were calculated using the World Health Organization Child Growth Standards Macro for SPSS. Underweight, stunting, and wasting were defined as WAZ < −2.0, HAZ < −2.0, and BMIZ < −2.0 standard deviations (SD), respectively.

### 2.3. Questionnaire

Self-designed structured questionnaires were administered to children's caregivers to obtain information. The questionnaire's contents mainly included age, sex, ethnicity, birth status, maternal anemia condition, G6PD deficiency, thalassemia, family status, and dietary habits. Trained interviewers conducted face-to-face interviews with the main caregivers. All caregivers participated on a voluntary basis and were not remunerated for their contribution.

### 2.4. Data Analysis

The information was recorded in a database using Excel. The data was cleaned and analyzed using Stata 16.0. Simple frequencies and percentages were used for categorical variables. Univariable conditional logistic regression was used to determine the association of independent variables with the dependent variable. Using a manual stepwise forward selection of variables, significant variables were put in the multivariable conditional logistic models, one at a time, to check for significant association until a final model of significant variables was achieved. A *P* value equal to or less than 0.05 was considered statistically significant.

## 3. Ethics Approval and Consent to Participate

This study was approved by the Institutional Review Board (IRB) of Shenzhen Pingshan Maternal and Child Health Hospital and complied with the national legislation and the Declaration of Helsinki guidelines. Informed written consent was obtained from the caregivers who agreed to participate in this study, and their participation was voluntary.

## 4. Results

### 4.1. Characteristics

A total of 552 children and 552 controls were enrolled in the study. There were 55.62% (307/552) males and 44.38% (245/552) females in both the case group and the control group. The mean ages for the cases and controls were 5.19 ± 0.80 years and 5.21 ± 0.79 years, respectively, and their age ranged between 3 and 7 years. There was no difference in the two matched variables including sex and age between the two groups. Greater than 90% of children, mothers, and fathers were of Han nationality in the two groups. The mean ages of the mothers were 32.77 ± 4.26 years and 32.42 ± 4.73 years for the case and control groups. The mean ages of the fathers were 34.86 ± 5.20 years and 32.99 ± 4.83 years for the case and control groups. More than 50% of children's parents had an educational level of senior high school or above in cases and controls (Table 1).

### 4.2. Univariable Analysis of Determinants of Anemia in Children Aged 3–7 years Old

Table 2 shows the results of univariable analysis conducted between anemia and each of the independent variables. Children in the East were likely to develop anemia than children in the West (OR = 1.86, 95% CI: 1.22∼2.86). Ethnic minority students and parents were prone to anemia compared with Han nationality (children: OR = 1.73, 95% CI: 1.02∼2.92; mother: OR = 1.76, 95% CI: 1.08∼2.88; father: OR = 1.92, 95% CI: 1.17∼3.14). Parents with higher education could decrease the risk of anemia in children (mother: OR = 0.84, 95% CI: 0.73∼0.97; father: 0.79, 95 CI: 0.69∼0.91). Maternal anemia before pregnancy or during pregnancy or nursing period could increase the risk of anemia in children (before pregnancy: OR = 8.57, 95% CI: 5.56∼13.19; pregnancy: OR = 6.04, 95% CI: 4.32∼8.45; nursing period: OR = 6.39, 95% CI: 4.54∼9.00). Mothers' active or passive exposure to smoke during pregnancy would increase the risk of anemia in children (OR = 2.64, 95% CI: 1.32∼5.28). Higher birth weight and height would reduce the risk of anemia in children (birth weight: OR = 0.53, 95% CI: 0.31∼0.92; birth height: OR = 0.92, 95% CI: 0.86∼0.98). Greater gestational age could reduce the risk of anemia in children (OR = 0.75, 95% CI: 0.63∼0.90). Mothers with spontaneous abortion could increase the risk of anemia in children (OR = 1.96, 95% CI: 1.28∼3.03). Children with G6PD deficiency or thalassemia could increase the risk of anemia in children (G6PD deficiency: OR = 6.00, 95% CI: 2.53∼14.24; thalassemia: OR = 48.00, 95% CI: 15.30∼151.58). Children having cold and cough in recent two previous weeks would increase the risk of anemia (OR = 1.42, 95% CI: 1.10∼1.83). Higher family income likely decreased the risk of anemia in children (OR = 0.80, 95% CI: 0.70∼0.91). Children eating for more than 30 min were prone to anemia (OR = 1.63, 95% CI: 1.28∼2.09). Children being picky eaters were likely to be anemic (OR = 2.2, 95% CI: 1.69∼2.87). Children who like eating snacks were more likely to become anemic (OR = 1.37, 95% CI: 1.03∼1.80). Similarly, children eating with no attention were likely to develop anemia (OR = 1.74, 95% CI: 1.33∼2.26).

Mothers' age, times of pregnancies, times of birth, mothers' consumption of tea during pregnancy, mode of delivery, diarrhea in recent weeks, trauma in the previous two weeks, permanent household population, consumption of dietary supplements containing iron, giving tea or milk to the children, cooking alone for the children, receiving childcare guidance, stunting, underweight, and wasting were not associated with anemia (*p* > 0.05).

### 4.3. Multivariable Analysis of Determinants of Anemia in Children Aged 3–7 years Old

The multivariable analysis (Table 3) identified an association between maternal anemia before or during pregnancy and lactation and more children having anemia (before pregnancy: OR = 2.14, 95% CI: 1.10 ∼ 4.15; during pregnancy: OR = 2.86, 95% CI: 1.66 ∼ 4.94; lactation: OR = 2.51, 95% CI: 1.13∼5.60). Mothers with greater gestational age could reduce the risk of anemia (OR = 0.72, 95% CI: 0.53∼0.96). G6PD deficiency or thalassemia in children was strongly associated with anemia in children (G6PD deficiency: OR = 8.12, 95% CI: 2.00∼33.44; thalassemia: OR = 36.25, 95% CI: 10.40∼126.43). Cold and cough in children in previous two weeks was significantly associated with an increased risk of anemia in children (OR = 1.56, 95% CI: 1.04∼2.34). Children with a higher income were less likely to have anemia (OR = 0.80, 95% CI: 0.65∼0.97). Children who were picky about food were prone to anemia (OR = 1.80, 95% CI: 1.20∼2.71).

## 5. Discussion

In China, the program for the development of children was implemented by the government and, has been working to solve the problem of anemia in children. Under this background, identifying the risk factors related to anemia is a very important task for eliminating anemia. Analytic research, such as case-control studies, could provide more reliable predictors to guide the prevention and control of anemia in children.

Maternal anemia was consistently related to the occurrence of childhood anemia. The study showed that children whose mothers had anemia before or during pregnancy and nursing period were more likely to develop anemia than those whose mothers did not have anemia during that time. This finding was consistent with the results from Leite MS [15], as mothers and children were most often mutually exposed to a common set of physical, socioeconomic, and dietary conditions. Besides, Catherine Smith [16] suggested that maternal anemia would increase the risk of preterm birth. Children born prematurely have a higher risk of anemia in childhood. Similarly, we discovered that mothers with greater gestational weeks could decrease the risk of anemia in children.

We also found that genetic diseases, such as thalassemia and G6PD deficiency, might increase the risk of anemia in children. In our study, children with thalassemia had the highest risk of developing anemia, with odds ratio of 34.26. Children with G6PD deficiency also had an increased risk of anemia, with an odds ratio of 8.78. Similarly, a study from Philippe Joly [17] reported that these two kinds of genetic diseases were associated with children developing anemia. Because of enzyme genetic deficiency or gene mutation, these two genetic diseases may lead to varying degrees of hemolysis in children, resulting in anemia.

In addition, we found that children with cold and cough in the previous two weeks were prone to anemia. Children with recurrent respiratory infections are more susceptible to anemia [18]. Therefore, when children develop respiratory symptoms such as colds and coughs, parents should seek medical services in a timely manner and take effective intervention measures to reduce the occurrence of adverse effects.

We also discovered that children's family economic level and living habits were important factors of influencing anemia in children. In this study, the higher the family income was, the less likely children were to develop anemia. A similar study reported that the odds of children with a lower family income having iron deficiency anemia were 3 times higher than those of children in the highest family income group [19]. Moreover, children who were picky eaters were prone to anemia. A meta-analysis also reported that poor food diversity was an important predictor of anemia in children under 5 [12]. Therefore, children with higher economic families might be more easily to acquire diversified food, good health education, and develop good living habits that protect them from anemia.

There were some limitations to this study. First, we could not determine the prevalence of anemia, so we could not compare the status of subgroups. Second, some information bias may have occurred in this study because some data were obtained from past information. Third, the factors associated with different types of anemia were not identified in this study. Four, some variables (e.g., dietary intake) were not included because of the limited resources.

## 6. Conclusions

In this study, anemia was significantly associated with maternal anemia, gestational weeks, G6PD deficiency, thalassemia, cold and cough in the previous two weeks, family income, and being a picky eater. Deeper knowledge about the etiology of anemia in this region is essential to its proper treatment and prevention.

## Figures and Tables

**Table 1:** Baseline characteristics of children.

	Cases (n, %)	Control (n, %)	Total (n, %)
*Sex*			
Male	307 (55.62)	307 (55.62)	614 (55.62)
Female	245 (44.38)	245 (44.38)	490 (44.38)
Age of children (years)	5.19 ± 0.80	5.21 ± 0.79	5.20 ± 0.80

*Ethnicity of children*			
Han	513 (92.93)	529 (95.83)	1042 (94.38)
Minorities	39 (7.07)	23 (4.17)	62 (5.62)
*Ethnicity of mothers*			
Han	508 (92.03)	527 (95.47)	1035 (93.75)
Minorities	44 (7.97)	25 (4.53)	69 (6.25)
*Age of mothers (years)*	32.77 ± 4.26	32.42 ± 4.73	32.60 ± 4.50

*Mothers' education*			
Junior high school and below	196 (35.61)	164 (29.71)	360 (32.61)
High school and secondary specialized school	157 (28.44)	175 (31.70)	332 (30.07)
Junior college	136 (24.64)	126 (22.83)	262 (23.73)
Bachelor degree and above	63 (11.41)	87 (15.76)	150 (13.59)
Age of fathers (years)	34.86 ± 5.20	34.86 ± 5.20	34.92 ± 5.02

*Ethnicity of fathers*			
Han	505 (91.49)	527 (95.47)	1032 (93.48)
Minorities	47 (8.51)	25 (4.53)	72 (6.52)

*Fathers' education*			
Junior high school and below	168 (30.43)	128 (23.19)	296 (26.81)
High school and secondary specialized school	168 (30.43)	180 (32.61)	348 (31.52)
Junior college	117 (21.20)	110 (19.93)	227 (20.56)
Bachelor degree and above	99 (17.93)	134 (24.82)	233 (21.11)

**Table 2:** Univariable analysis of determinants of anemia in children.

	Cases (n, %)	Control (n, %)	*P*	OR (95% CI)
*Children's region*				
Western region	46 (8.33)	63 (11.41)		1
Central region	134 (24.28)	195 (35.33)	0.982	1.00 (0.65∼1.55)
Eastern region	372 (67.39)	294 (53.26)	0.004	1.86 (1.22∼2.86)

*Ethnicity of children*				
Han	513 (92.93)	529 (95.83)		1
Minorities	39 (7.07)	23 (4.17)	0.041	1.73 (1.02∼2.92)

*Ethnicity of mothers*				
Han	508 (92.03)	527 (95.47)		1
Minorities	44 (7.97)	25 (4.53)	0.024	1.76 (1.08∼2.88)
Age of mothers (years)	32.77 ± 4.26	32.42 ± 4.73	0.192	0.98 (0.96∼1.01)
Mothers' education			0.015	0.84 (0.73∼0.97)
Junior high school and below	196 (35.61)	164 (29.71)		
High school and secondary specialized school	157 (28.44)	175 (31.70)		
Junior college	136 (24.64)	126 (22.83)		
Bachelor degree and above	63 (11.41)	87 (15.76)		

*Ethnicity of fathers*				
Han	505 (91.49)	527 (95.47)		1
Minorities	47 (8.51)	25 (4.53)	0.010	1.92 (1.17∼3.14)
Fathers' education			0.001	0.79 (0.69∼0.91)
Junior high school and below	168 (30.43)	128 (23.19)		
High school and secondary specialized school	168 (30.43)	180 (32.61)		
Junior college	117 (21.20)	110 (19.93)		
Bachelor degree and above	99 (17.93)	134 (24.82)		

*Times of pregnancy*				
<3	401 (72.64)	426 (77.17)		1
≥3	151 (27.36)	126 (22.83)	0.076	1.30 (0.97∼1.71)

*Times of birth*				
1	183 (33.15)	200 (36.23)		1
2	310 (56.16)	301 (54.53)	0.322	1.14 (0.88∼1.48)
≥3	59 (10.69)	51 (9.24)	0.245	1.30 (0.83∼2.03)

*Maternal anemia before pregnancy*				
Yes	221 (40.04)	47 (8.51)	<0.001	8.57 (5.56∼13.19)
No	331 (59.96)	505 (91.49)		1

*Maternal anemia during pregnancy*				
Yes	290 (52.06)	87 (15.62)	<0.001	6.04 (4.32∼8.45)
No	267 (47.94)	470 (84.38)		1

*Maternal anemia during nursing period*				
Yes	290 (52.54)	85 (15.40)	<0.001	6.39 (4.54∼9.00)
No	262 (47.46)	467 (84.60)		1

*Mothers smoked actively or passively during pregnancy*				
Yes	32 (5.80)	14 (2.54)	0.006	2.64 (1.32∼5.28)
No	520 (94.20)	538 (97.46)		

*Mothers drank tea during pregnancy*				
Yes	44 (7.97)	32 (5.80)	0.154	1.41 (0.88∼2.27)
No	508 (92.03)	520 (94.20)		
Birth weight (g)			0.023	0.53 (0.31∼0.92)
<2500	14 (2.54)	11 (1.99)		
2500∼4000	528 (95.65)	516 (93.48)		
>4000	10 (1.81)	25 (4.53)		
Birth height (cm)	50.07 ± 2.02	50.32 ± 1.84	0.017	0.92 (0.86∼0.98)
Gestational weeks			0.002	0.75 (0.63∼0.90)
<37	31 (5.62)	21 (3.80)		
37∼38	149 (26.99)	117 (21.20)		
39∼40	320 (57.97)	342 (61.96)		
>40	52 (9.42)	72 (13.04)		

*History of spontaneous abortion of mother*				
Yes	69 (12.50)	39 (7.07)	0.002	1.96 (1.28∼3.03)
No	483 (87.50)	513 (92.93)		

*Mode of delivery*				
Spontaneous labor	379 (68.66)	355 (64.31)		1
Cesarean section	173 (31.34)	197 (35.69)	0.116	0.81 (0.63∼1.05)

G6PD deficiency of children				
Yes	38 (6.88)	8 (1.45)	<0.001	6.00 (2.53∼14.24)
No	514 (93.12)	544 (98.55)		1

*Thalassemia of children*				
Yes	146 (26.45)	5 (0.91)	<0.001	48.00 (15.30∼151.58)
No	406 (73.55)	547 (99.09)		1

*Diarrhea in recent weeks*				
Yes	6 (1.09)	4 (0.72)	0.530	1.50 (0.42∼5.32)
No	546 (98.91)	548 (99.28)		1

*Cold and cough in previous two weeks*				
Yes	244 (44.20)	201 (36.41)	0.006	1.42 (1.10∼1.83)
No	308 (55.80)	351 (63.59)		1

*Trauma in previous two weeks*				
Yes	4 (0.72)	1 (0.18)	0.215	4.00 (0.45∼35.79)
No	548 (99.28)	551 (99.82)		1

*Permanent household population*				
≤4	264 (47.83)	243 (44.02)		
>4	288 (52.17)	309 (55.98)	0.209	0.86 (0.68∼1.09)

Family income (RMB/moth)			0.001	0.80 (0.70∼0.91)
<3000	50 (9.06)	32 (5.80)		
3000∼5999	220 (39.86)	191 (34.60)		
6000∼8999	105 (19.02)	115 (20.83)		
≥9000	177 (32.07)	214 (38.77)		

*Consumption of iron supplements*				
Yes	272 (49.28)	288 (52.17)		1
No	280 (50.72)	264 (47.83)	0.316	1.13 (0.89∼1.45)

Giving tea to the children				
Yes	41 (7.43)	37 (6.70)	0.642	1.11 (0.71∼1.76)
No	511 (92.57)	515 (93.30)		1

Giving milk to the children				
Yes	499 (90.40)	506 (91.67)	0.448	0.85 (0.55∼1.30)
No	53 (9.60)	46 (8.33)		1

*Cooking alone for the children*				
Yes	386 (69.93)	395 (71.56)		1
No	166 (30.07)	157 (28.44)	0.539	1.09 (0.83∼1.42)

*Children eating for more than 30 min*				
Yes	325 (58.88)	259 (46.92)	<0.001	1.63 (1.28∼2.09)
No	227 (41.12)	293 (53.08)		1

*Picky eater*				
Yes	385 (69.75)	289 (52.63)	<0.001	2.2 (1.69∼2.87)
No	167 (30.25)	263 (47.64)		1

*Like eating snacks*				
Yes	418 (75.72)	386 (69.93)	0.026	1.37 (1.03∼1.80)
No	134 (24.28)	166 (30.07)		

*Eating with no attention*				
Yes	408 (73.91)	344 (62.32)	<0.001	1.74 (1.33∼2.26)
No	144 (26.09)	208 (37.68)		

*Receiving childcare guidance*				
Yes	223 (40.40)	249 (45.11)		1
No	329 (59.60)	303 (54.89)	0.105	1.23 (0.96∼1.57)
Stunting			0.060	1.7 (0.98∼2.95)
Yes	39 (7.07)	25 (4.53)		
No	513 (92.93)	527 (95.47)		
Underweight			0.213	0.63 (0.31∼1.30)
Yes	12 (2.17)	19 (3.44)		
No	540 (97.83)	533 (96.56)		
Wasting			0.198	1.64 (0.77∼3.46)
Yes	19 (3.44)	12 (2.17)		
No	533 (96.56)	540 (97.83)		

**Table 3:** Multivariable analysis of determinants of anemia in children.

Variables	b	SE	*P*	OR (95% CI)
Maternal anemia before pregnancy	0.76	0.34	0.025	2.14 (1.10∼4.15)
Maternal anemia during pregnancy	1.05	0.28	<0.001	2.86 (1.66∼4.94)
Maternal anemia during nursing period	0.92	0.41	0.024	2.51 (1.13∼5.60)
Gestational weeks	-0.34	0.15	0.024	0.72 (0.53∼0.96)
G6PD deficiency	2.10	0.72	0.003	8.12 (2.00∼33.04)
Having thalassemia	3.59	0.64	<0.001	36.25 (10.40∼126.43)
Cold and cough in previous two weeks	0.45	0.21	0.031	1.56 (1.04∼2.34)
Family income (RMB/month)	-0.22	0.10	0.026	0.80 (0.65∼0.97)
Picky eater	0.59	0.21	0.005	1.80 (1.20∼2.71)

## Data Availability

The data used to support the findings of this study are available from the corresponding author upon request.

## Abbreviations

OR: Odds ratio
CI: Confidence interval
G6PD: Glucose-6-phosphate dehydrogenase.

## References

[1] McLean E., Cogswell M., Egli I., Wojdyla D., de Benoist B. (2009). Worldwide prevalence of anaemia, WHO vitamin and mineral nutrition information system, 1993-2005. *Public Health Nutrition*.

[2] Lopez A., Cacoub P., Macdougall I. C., Peyrin-Biroulet L. (2016). Iron deficiency anaemia. *Lancet*.

[3] Pivina L., Semenova Y., Doşa M. D., Dauletyarova M., Bjørklund G. (2019). Iron deficiency, cognitive functions, and neurobehavioral disorders in children. *Journal of Molecular Neuroscience*.

[4] Bailey R. L., West K. P. (2015). Black RE: the epidemiology of global micronutrient deficiencies. *Annals of Nutrition & Metabolism*.

[5] Haas J. D., Brownlie Tt (2001). Iron deficiency and reduced work capacity: a critical review of the research to determine a causal relationship. *Journal of Nutrition*.

[6] Balarajan Y., Ramakrishnan U., Özaltin E., Shankar A. H., Subramanian S. V. (2011). Anaemia in low-income and middle-income countries. *The Lancet*.

[7] Wu J., Hu Y., Li M. (2019). Prevalence of anemia in Chinese children and adolescents and its associated factors. *International Journal of Environmental Research and Public Health*.

[8] Liu J., Huo J., Liu Z., Sun J., Huang J. (2021). Prevalence and temporal trend (2016-2018) of anaemia among 6-23-month-old infants and young children in China. *International Journal of Environmental Research and Public Health*.

[9] Du W., Zhang B. (2008). Study on anemia status and risk factors in children under 5 years old. *Journal of Hygiene Research*.

[10] Li H., Xiao J., Liao M. (2020). Anemia prevalence, severity and associated factors among children aged 6-71 months in rural Hunan Province, China: a community-based cross-sectional study. *BMC Public Health*.

[11] Allali S., Brousse V., Sacri A. S., Chalumeau M., de Montalembert M. (2017). Anemia in children: prevalence, causes, diagnostic work-up, and long-term consequences. *Expert Review of Hematology*.

[12] Belachew A., Tewabe T. (2020). Under-five anemia and its associated factors with dietary diversity, food security, stunted, and deworming in Ethiopia: systematic review and meta-analysis. *Systematic Reviews*.

[13] Bing-bing G. U. O., Ya-rong W. E. I., Jing-jing P. E. I. (2018). Influencing factors of nutritional anemia in 0-6 years old children in China: a meta-analysis. *Chinese Journal of Public Health*.

[14] Kassebaum N. J. (2016). The global burden of anemia. *Hematology-Oncology Clinics of North America*.

[15] Leite M. S., Cardoso A. M., Coimbra C. E. A. (2013). Prevalence of anemia and associated factors among indigenous children in Brazil: results from the First National Survey of Indigenous People’s Health and Nutrition. *Nutrition Journal*.

[16] Smith C., Teng F., Branch E. (2019). Maternal and perinatal morbidity and mortality associated with anemia in pregnancy. *Obstetrics & Gynecology*.

[17] Joly P., Garnier N., Kebaili K. (2016). G6PD deficiency and absence of *α*-thalassemia increase the risk for cerebral vasculopathy in children with sickle cell anemia. *European Journal of Haematology*.

[18] Xiao G. A. O., Yan Y. A. N., Xiang S. (2017). Influential factors of iron deficient anemia among infants aged 8 months based on a case-control study. *J Cent South Univ (Med Sci)*.

[19] Bayoumi I., Parkin P. C., Birken C. S., Maguire J. L., Borkhoff C. M. (2020). Association of family income and risk of food insecurity with iron status in young children. *JAMA Network Open*.

